# Non-immediate drug hypersensitivity reactions secondary to intravitreal anti-vascular endothelial growth factors

**DOI:** 10.1007/s00417-021-05353-3

**Published:** 2021-09-16

**Authors:** E. Moret, A. Ambresin, C. Gianniou, J. Bijon, C. Besse-Hayat, S. Bogiatzi, D. Hohl, F. Spertini, I. Mantel

**Affiliations:** 1 Department of Ophthalmology, Jules-Gonin Eye Hospital, University of Lausanne, Foundation Asile des Aveugles, Lausanne, Switzerland; 2 Department of Medicine, Division of Dermatology and Venereology, Lausanne University Hospital CHUV, Lausanne, Switzerland; 3 Department of Medicine, Division of Immunology and Allergy, Lausanne University Hospital CHUV, Lausanne, Switzerland

**Keywords:** Intravitreal anti-VEGF, Aflibercept, Ranibizumab, Bevacizumab, Drug hypersensitivity reaction, Cutaneous adverse events

## Abstract

**Purpose:**

To describe a series of non-immediate drug hypersensitivity reactions after intravitreal anti-vascular endothelial growth factors (anti-VEGFs).

**Patients and methods:**

Retrospective report of 6 patients with cutaneous non-immediate drug hypersensitivity reactions following intravitreal anti-VEGF injections, 4 after ranibizumab, 1 after bevacizumab and 1 after aflibercept.

**Results:**

Clinical manifestations ranged from mild maculopapular rash, purpura to severe generalized erythroderma, with or without systemic involvement such as microscopic hematuria and proteinuria or fever. In two out of the six patients, reintroduction of either the same or an alternative anti-VEGF drug did induce a recurrence of the drug hypersensitivity reaction, while 4 patients showed no recurrence.

**Conclusion:**

Cutaneous non-immediate drug hypersensitivity reactions secondary to intravitreal anti-VEGF may occur. Continuation of the same drug or switch to another anti-VEGF may either induce recurrence or be well supported by the patient. The decision of drug discontinuation should be guided by the severity of the disease.


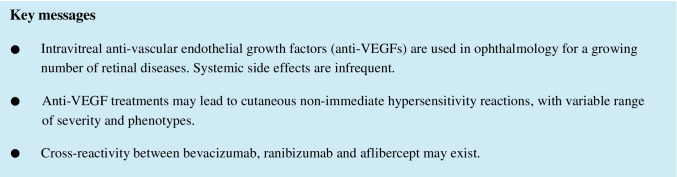


## Introduction

From 2006, intravitreal anti-vascular endothelial growth factors (anti-VEGFs) have become the standard treatment in ophthalmology, which has been shown to be effective in a growing number of retinal diseases.

Anti-VEGF molecules are directed against the action of VEGF-A, a mediator protein promoting intraocular angiogenesis and vascular permeability. Different isoforms of VEGF-A exist: VEGF206, VEGF189, VEGF165 and VEGF121 [1], where VEGF165 is the isoform that is mainly responsible for neovascularization and leakage in retinal diseases [2, 3].

**Fig. 1:**
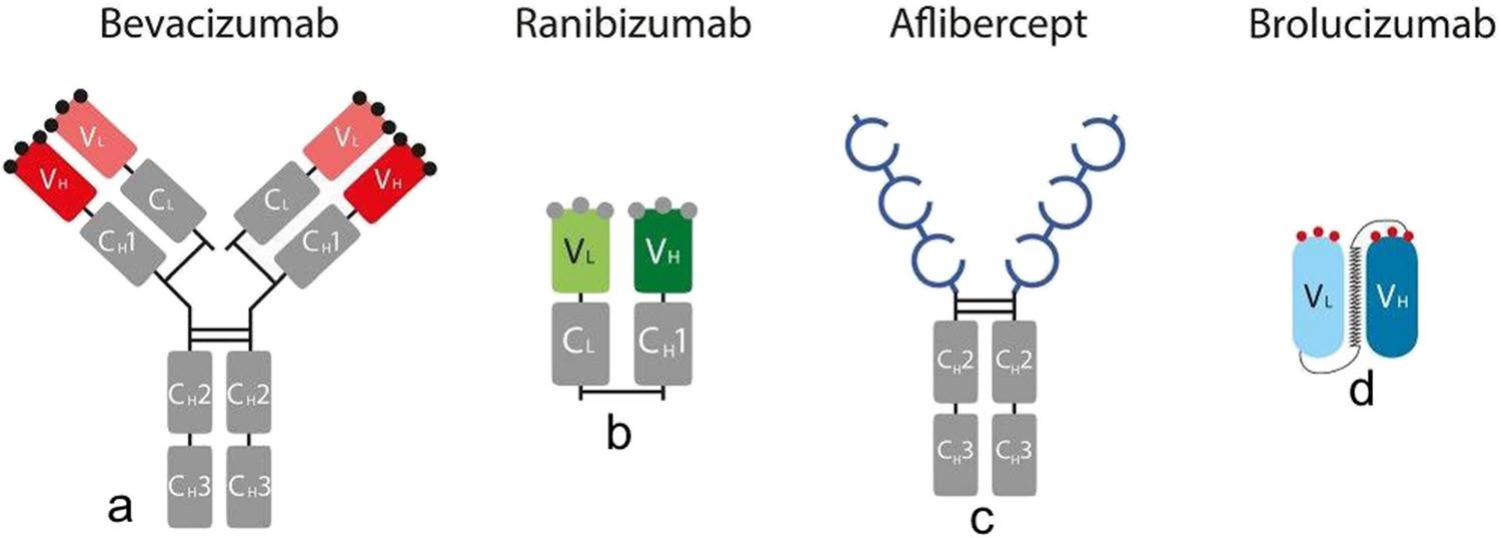
Schematic structure of bevacizumab (**a**), ranibizumab (**b**), aflibercept (**c**) and brolucizumab (**d**). Bevacizumab is a full-length humanized IgG1 mAb. Ranibizumab is composed of the high-affinity recombinant Fab-portion of the IgG1 mAb of bevacizumab, devoid of the Fc-component. Aflibercept is composed from the fusion of domains of VEGF-receptor 1 and 2 to an IgG1 Fc-portion. Brolucizumab is a single-chain antibody fragment (scFv) composed of two complementarity-determining regions of the anti-VEGF molecule grafted to a human scFv scaffold

Four anti-VEGFs are currently used for intravitreal treatment: bevacizumab, ranibizumab, aflibercept and brolucizumab, the four of them differing in their molecular structure (Fig. 1). Bevacizumab is a fully humanized immunoglobulin G1 (IgG1) kappa isotype monoclonal antibody (mAb) of 148 kDa that binds all isoforms of VEGF-A [4]. Ranibizumab is a 48 kDa high-affinity recombinant IgG1 kappa mAb fragment composed with the fragment antigen-binding (Fab) portion of the antibody, devoid of the fragment crystallizable (Fc) region, that is derived from the same humanized murine antibody than bevacizumab [3, 4]. Thus, bevacizumab and ranibizumab include a relatively similar Fab-portion. Both of them neutralize all isoforms of VEGF-A. However, aflibercept is a soluble decoy receptor of 115 kDa, manufactured by the fusion of the second binding domain of the native VEGF receptor 1 and the third binding domain of the VEGF receptor 2 to the Fc-portion of human IgG1. It has a higher binding affinity for all VEGF isoforms than the native receptors [2, 5]. Although it contains an Fc-domain similar to bevacizumab, there is no Fab-portion such as present in bevacizumab or ranibizumab. Recently, a new anti-VEGF molecule has been introduced to the market: brolucizumab is a single-chain antibody fragment (scFv), the smallest functional subunit of an antibody, which was developed by grafting two complementarity-determining regions of the anti-VEGF molecule to a human scFv scaffold. It has a smaller molecular weight of 26 kDa and binds all isoforms of VEGF-A [6].

Current indications for anti-VEGF intravitreal therapy (IVT) include choroidal neovascularizations (CNV) due to age related macular degeneration (AMD), pathologic myopia and CNV of other origin, macular edema secondary to diabetes and retinal vein occlusion.

Safety of intravitreal anti-VEGF injections has been investigated not only in the pivotal trials but also from real life practice [7, 8]. Most frequently reported ocular side effects include corneal abrasion, ocular haemorrhages, intraocular pressure elevation, cataract, rhegmatogenous retinal detachment, retinal pigment epithelium tear, intraocular inflammation and endophthalmitis [7, 8]. All of these side effects are procedure related rather than drug related.

The anti-VEGF agents themselves appear to be very well tolerated. There are no confirmed drug-related ocular side effects, although recent reports suggest the occurrence of sterile endophthalmitis to aflibercept [9] and intraocular inflammation, vasculitis and vascular occlusion following brolucizumab intravitreal injection [10].

Systemically, there is an ongoing debate whether intravitreal use of anti-VEGF does or does not increase the risk of arterial hypertension and thromboembolic events (stroke and myocardial infarct) [11].

Cutaneous eruptions have occasionally been reported in conjunction with intravitreal anti-VEGF, such as de novo cutaneous lupus erythematosus, acute generalized exanthematous pustulosis, head and trunk papulopustular eruption, maculopapular rash, facial skin redness and itchy diffuse rash [12–17]. The initial pivotal trials did not report cutaneous eruptions as anti-VEGF related side effects.

The aim of this paper was to describe an additional case series of these rare cutaneous non-immediate hypersensitivity reactions after anti-VEGF treatment, and to present the current presumed pathophysiology mechanism underlying these reactions.

## Methods

This retrospective study of a consecutive case series was performed in accordance with the tenets of the ethical standards of the Ethics Committee of the Swiss Federal Department of Health (no. CER-VD 19/15 and 20/15), the 1964 Declaration of Helsinki, and its later amendments. No informed consent was required.

We identified patients who presented cutaneous non-immediate hypersensitivity reaction following intravitreal injection with anti-VEGF.

The clinical files were reviewed, and the following data were extracted for the study: retinal indication for intravitreal anti-VEGF therapy, the anti-VEGF agent used, cutaneous manifestations and time delay between injection and first symptoms, duration of symptoms, dermatological or immunological tests, need for dermatological treatment, the ophthalmic treatment strategy following the cutaneous event and any cutaneous recurrence.

The routine ophthalmic care protocol included for the initial diagnosis a clinical evaluation, optical coherence tomography (OCT) and fluorescein/indocyanine green angiographies. As indicated by the diagnosis, the treatment protocol consisted of 3 initial loading doses of anti-VEGF in monthly intervals, followed by OCT guided retreatment (pro re nata until 2013, observe and plan [18] from 2013).

The data is presented in a descriptive way. The number of patients does not allow for statistical association analyses.

## Results

Between January 2010 and December 2018, approximately 55,000 anti-VEGF IVTs have been performed in our Department of Ophthalmology, University of Lausanne, Jules Gonin Eye Hospital, Switzerland. Within this time span, 5 events of cutaneous non-immediate hypersensitivity reactions were seen. A sixth patient was reported to us from a private ophthalmologist.

Table 1 gives a systematic overview of the patients’ characteristics. Individual descriptions with more details are given below.

**Table 1:** Summary for clinics and additional test results for patients 1 to 6

Characteristic	Patient 1	Patient 2	Patient 3	Patient 4	Patient 5	Patient 6
Age at presentation (years old)	66	81	83	93	81	65
Retinal disease justifying anti-VEGF IVT	nAMD	BRVO	nAMD	nAMD	nAMD	nAMD
Incriminated anti-VEGF	RNB	BVZ	RNB	RNB	AFL	RNB
IVT number	4	1	2	25	1	6
Cutaneous manifestation (with localisation)	Pruriginous erythema (neck, upper back, shoulders, upper limbs)	Pruritic erythematous maculopapular rash (face)	Generalized erythroderma	Palpable purpura (both legs)	Maculopapular rash (both legs)	Painful pruritic erythematous swelling (both ankles and feet)
Systemic manifestation	None	Fever	None	Microscopic hematuria and proteinuria	None	Upper and lower lips aphtas, feverishness and shivers
Degree of severity	Moderate	Mild	Severe	Moderate	Mild	Mild
Time of onset after IVT	4 days	3 days	4 weeks	48 h	3 days	4 days
Symptoms duration	2 weeks	10 days	8 weeks	10 days	1 month	3 weeks
Skin biopsy result	NA	NA	Eosinophilic spongiosis dermatitis with negative immunofluorescence	Leucocytoclastic vasculitis with IgA deposits	NA	Superficial dermatitis with dermal swelling and granular C3 deposits in the vessel’s walls on direct immunofluorescence
Use of an alternative anti-VEGF after NIDHRs	AFL	BVZ, RNB	AFL	AFL	AFL	AFL
Recurrence of NIDHRs after anti-VEGF reintroduction	Yes (pruritus 3 days after exposure to AFL)	No (reexposure to BVZ, exposure to RNB)	Yes (recurrence after reexposure to RNB, and after exposure to AFL)	No (exposure to AFL)	No (reexposure to AFL)	No (exposure to AFL)

*anti-VEGF* anti-vacular endothelial growth factor, *IVT* intravitreal therapy, *nAMD* neovascular age-related macular degeneration, *BRVO* branch retinal vein occlusion, *BVZ* bevacizumab, *RNB* ranibizumab, *AFL* afibercept, *NA* not applicable, *NIDHRs* non-immediate drug hypersensitivity reactions

### Patient 1

A 66-year-old woman, known for exudative type 1 neovascularisation due to age-related macular degeneration in her right eye, was successfully treated with intravitreal ranibizumab injections. However, four days after the forth injection, she developed a pruriginous erythema on the neck, on the upper region of the back, on her shoulders and upper limbs down to the elbows on both sides. She was treated with topical corticoids (clobetasone) together with oral antihistamine (cetirizine). Two weeks later, the cutaneous lesions evolved into post-inflammatory desquamation flaps with persisting peripheral clinically inflammatory margins. An extensive questionnaire revealed no recent unusual drug intake, no infection sign and no unusual cosmetic use in the previous three months.

Cutaneous tests (prick tests, intradermal tests and patch tests) were performed for the various substances used during intravitreal injections procedure, either routinely or in exceptional cases (tetracaine, oxybuprocaine, chlorhexidine, benzalkonium chlorure, povidone iodine, tobramycine and dexamethasone, procaine, lidocaine, benzocaine, latex, ranibizumab and bevacizumab). None of these substances induced a significant cutaneous reaction.

Based on the clinical history and presentation, and despite the negative cutaneous tests, the most likely diagnosis was considered to be a type III hypersensitivity reaction secondary to ranibizumab.

Because of a high risk for recurrences after such an immune reaction, it was strongly recommended to avoid any future exposure to ranibizumab. In addition, bevacizumab was discarded due to the similarity of the Fab fragment with ranibizumab.

An exudative reactivation of the neovascular AMD with visual acuity loss occurred 2 years later, and was successfully treated with an intravitreal injection of aflibercept. The patient reported only low-grade skin itching three days after the injection without further systemic involvement. Fortunately, no further injections were required.

### Patient 2

An 81-year-old woman was referred to our medical retina department for branch retinal vein occlusion in her left eye. The associated cystoid macular edema was treated with intravitreal bevacizumab injections. Three days after the first injection, and two days after the second one, respectively, the patient developed a ten-days-lasting pruritic erythematous maculopapular rash on the face associated with fever. Based on the clinical history and description of skin eruption, drug induced type III hypersensitivity reaction was highly suspected.

Cutaneous tests (prick tests and intradermal tests) were performed, testing for local anaesthetics and desinfecting solutions (tetracaine, oxybuprocaine, proxymetacaine, chlorhexidine). A positive skin reaction to tetracaine and oxybuprocaine was found. A third bevacizumab injection was performed before the skin test results were available, and the patient did not notice any cutaneous side effect. However, after interdisciplinary discussion, a switch to a different anti-VEGF drug was recommended. Ranibizumab was chosen for the following two injections, and no further cutaneous reactions were observed.

### Patient 3

An 83-year-old man was followed by our department for neovascular age-related macular degeneration with type 2 neovascularization in his left eye.

Four weeks after a second intravitreal ranibizumab injection, the patient developed a generalized erythroderma, with diffuse pruritic erythematous cutaneous eruptions. The third ranibizumab injection was uneventful, but the fourth injection of ranibizumab was followed by a recurrence of cutaneous symptoms 4 weeks later.

**Fig. 2:**
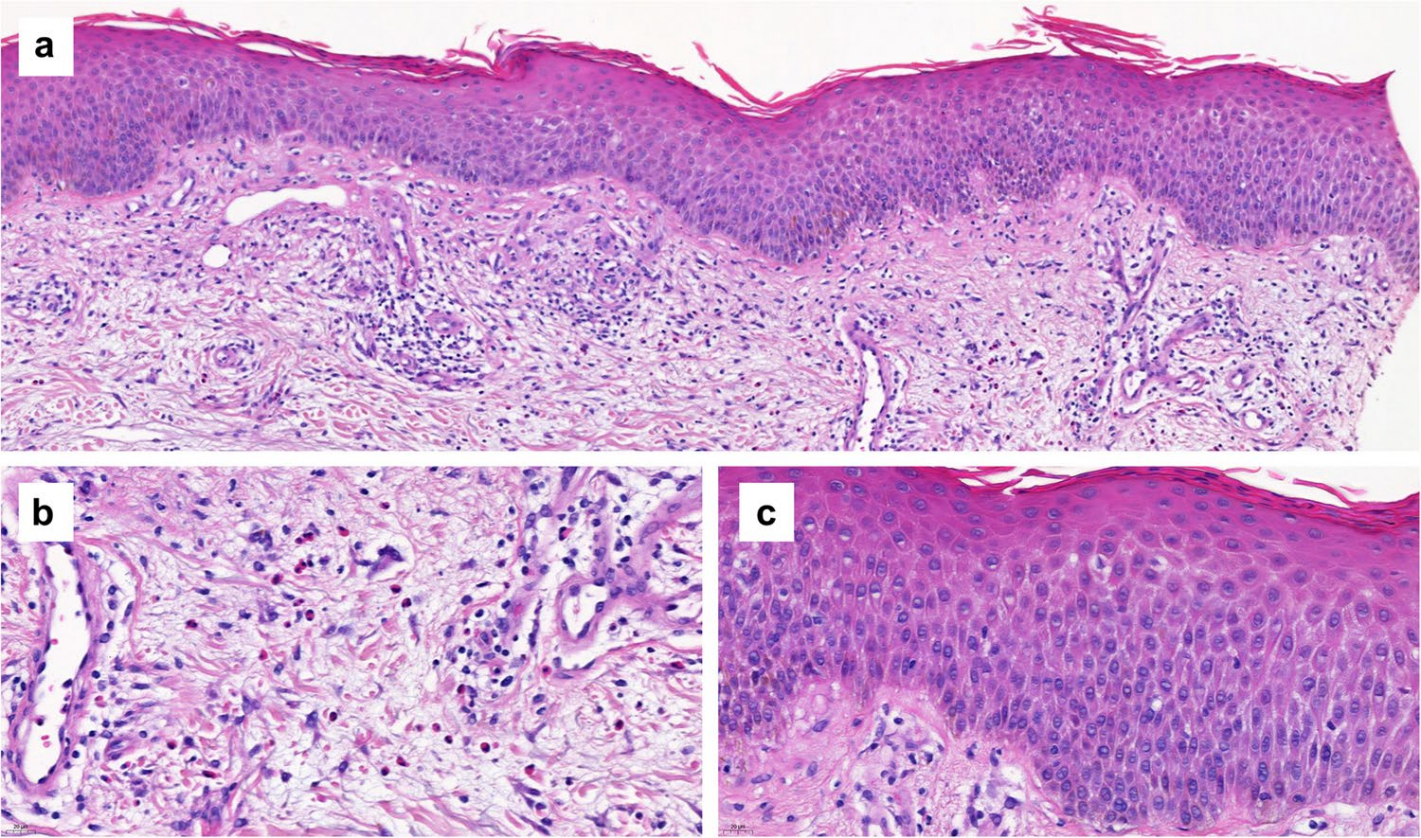
**a** Skin biopsy of patient 3: Spongiotic dermatitis with eosinophils. Hematoxylin–eosin staining of a formalin fixed skin biopsy showing epidermal spongiosis (**b**) and a perivascular infiltrate with numerous eosinophils (**c**). The vascular walls of the superficial plexus show a discrete inflammation but no signs of destruction

Skin biopsy showed a eosinophilic spongiotic dermatitis with negative immunofluorescence (Fig. 2), compatible with a type III hypersensitivity reaction. Topical corticosteroids (clobetasol cream) and a topical immunosuppressive treatment (tacrolimus cream 0.1%) were prescribed, followed by rapid improvement of the skin lesions.

The treatment was then switched to aflibercept. Nevertheless, three weeks after aflibercept, the patient showed an even stronger erythematous cutaneous eruption.

Finally, anti-VEGF therapy was halted and the treatment for neovascular AMD was switched to photodynamic therapy with verteporfine.

Based on the clinical history and on the skin biopsy, the diagnosis of delayed-type drug-induced cutaneous reaction to intravitreal anti-VEGFs was made.

### Patient 4

**Fig. 3:**
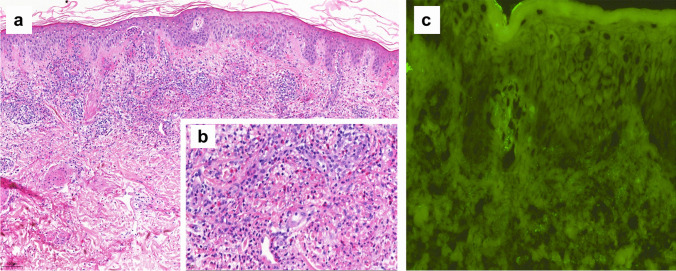
Skin biopsy of patient 4: Leukocytoclastic vasculitis histopathology. **a** Hematoxylin–eosin staining of a formalin fixed skin biopsy showing a dense perivascular neutrophil infiltrate with nuclear dust and purpura (10× magnification). The vascular walls of the superficial plexus show marked destruction (**b** 40× magnification) with thickened eosinophilic walls, fibrin deposition and inflammatory cell infiltration. **c** IgA vasculitis direct immunofluorescence. Granular IgA deposition within the walls of superficial dermal vessels (10× magnification)

A 93-year-old woman was treated in our department with intravitreal ranibizumab injections for neovascular age-related macular degeneration with type 3 neovascularization in her right eye. Forty-eight hours after the 25^th^ injection, the patient developed palpable purpura on both legs, associated with microscopic hematuria and proteinuria, without renal failure. A skin biopsy showed a leucocytoclastic vasculitis with IgA deposits (Fig. 3). It was considered to be a type III hypersensitivity reaction, most likely due to ranibizumab, in the absence of other possible etiologies and because of the temporal correlation between drug administration and the cutaneous complication.

As the likelihood of ranibizumab-related vasculitis was high, the ophthalmic treatment was changed to aflibercept. No further drug-related side effects were observed for the following 10 injections.

### Patient 5

**Fig. 4:**
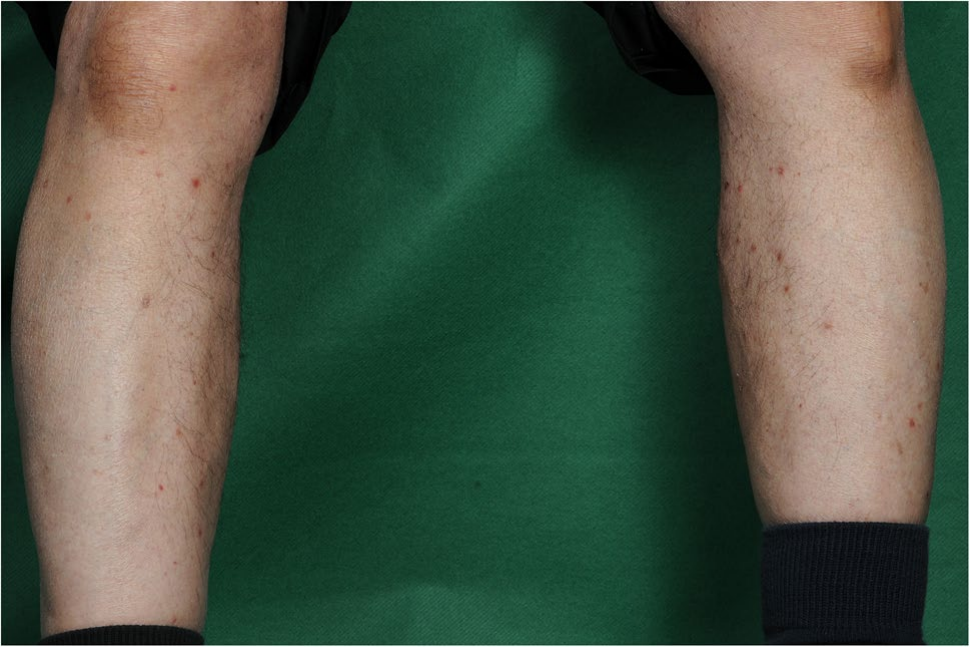
Skin lesions of patient 5: maculopapular rash on both legs

An 81-year-old man suffering from haemorrhagic neovascular age-related macular degeneration with type 1 and type 2 choroidal neovascularizations in his right eye underwent treatment with intravitreal aflibercept. Three days after the first injection, he developed a maculopapular rash on both legs, which resolved after one month (Fig. 4). An aflibercept-related drug eruption was suspected. However, due to the relative weak cutaneous reaction, the anti-VEGF treatment was continued using aflibercept according to need. No further cutaneous eruption were observed after the following 4 injections. The patient moved to another country and was lost to further follow-up.

### Patient 6

A 65-year-old woman with neovascular AMD underwent intravitreal ranibizumab treatment. The first 5 injections were uneventful. However, four days after her sixth injection, she developed painful pruritic erythematous swelling of both ankles and feet, associated with upper and lower lips aphtas, feverishness and shivers. Antibiotics (amoxicillin/clavulanic acid) were given for suspected bilateral ankles dermohypodermitis, but did not improve the situation. Blood tests showed an eosinophilic inflammatory syndrome.

**Fig. 5:**
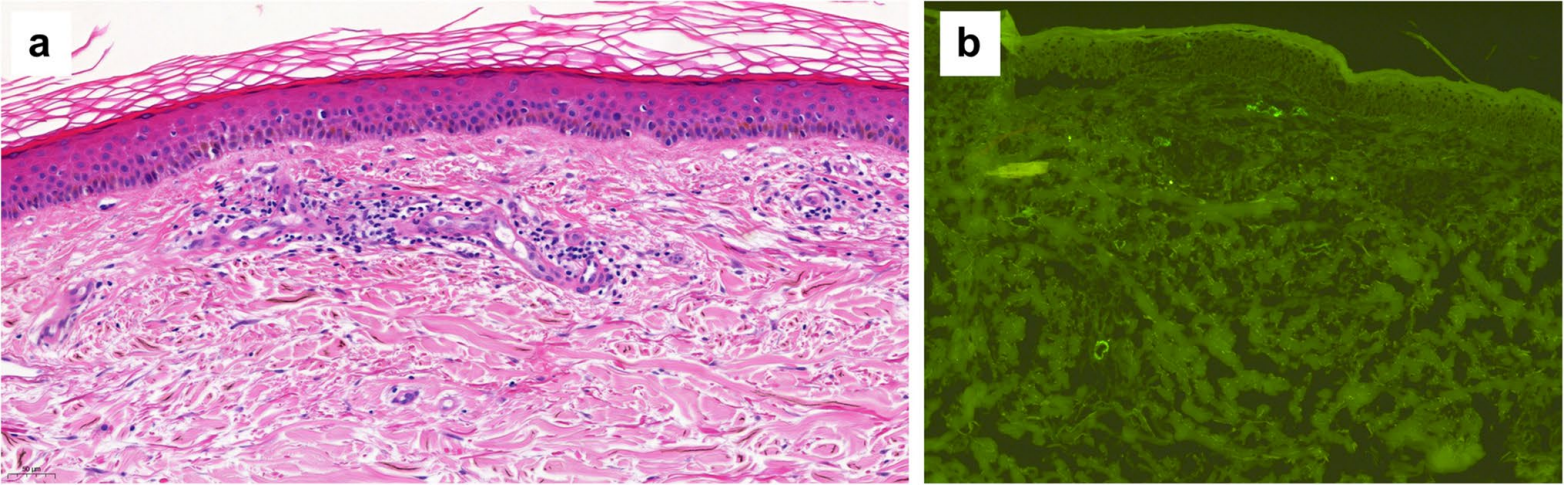
Skin biopsy of patient 6: Leukocytoclastic vasculitis histopathology. **a** Hematoxylin–eosin staining of a formalin fixed skin biopsy showing a perivascular neutrophil infiltrate with nuclear dust and discret purpura. The vascular walls of the superficial plexus are thickened with fibrin deposition and inflammatory cell infiltration (10× magnification). **b** Vascular inflammation. Direct immunofluorescence showing granular C3 deposition within the walls of superficial dermal vessels (10× magnification)

A skin biopsy was performed, showing a superficial dermatitis with dermal swelling and granular C3 deposits in the vessel’s walls on direct immunofluorescence (Fig. 5). A type III hypersensitivity reaction to ranibizumab was the most likely interpretation.

The symptoms and the inflammatory parameters rapidly improved on oral prednisone. Only some mild pretibial petechial lesions and moderate desquamation were found five days after corticoid treatment.

For further anti-VEGF treatment, the treatment drug was switched to aflibercept. No further complications were reported.

## Discussion

Our small case series of type III hypersensitivity reactions to intravitreal anti-VEGF illustrates the existence of such reaction, and the variability of its severity. Due to sometimes more severe immunological systemic reactions, recognition of the causal relationship with the anti-VEGF drug may be crucial in order to adjust the ophthalmic treatment strategy.

We describe immunologic reactions following bevacizumab, ranibizumab and aflibercept. In all cases, no concomitant intraocular inflammation or vasculitis was associated with these cutaneous manifestations. Previous anecdotal reports have reported similar reactions to all of these three drugs [12–17]. However, so far no similar event is known after brolucizumab, the most recent anti-VEGF drug for intraocular use.

As described in the introduction, the molecular structure of bevacizumab, ranibizumab and aflibercept are quite different although they partially share some similar fragments. All of them are part of the biologic agents’ drug class, although some bio-engineering process plays an important role in their production as well. Antibodies are typical biologic agents. They are used in a large range of systemic diseases, including chronic inflammatory and autoimmune diseases. Antibodies of any sort may provoke non-immediate drug hypersensitivity reactions (NIDHRs) [19–21]. Immune-mediated adverse events to systemic administration of antibodies are well known, but not so after intraocular administration. Bevacizumab, ranibizumab, and aflibercept share some characteristics with the typical antibodies: bevacizumab is the only entire antibody structure, while ranibizumab is only a Fab fragment, and aflibercept has a Fc antibody fragment.

IDHRs are a subtype of unpredictable drugs’ side effects, independent of their direct pharmacological action. In contrast with IgE-mediated immediate drug hypersensitivity reactions (IDHRs) (type I hypersensitivity reaction—IgE mediated—, according to Gell and Coombs classification system of immune reaction), which occur mostly within one hour after the drug exposure, NIDHRs (type II—IgG/IgM cytotoxic reactions –, type III—IgM/IgG immune complexes mediated—and type IV—T-cell mediated—hypersensitivity reactions) are antibody or cell-mediated reactions and arise usually days or weeks after initial drug administration. Typically, it affects the skin, although fever, haematological reaction, joints, lung, liver and kidney may occur [22].

In this retrospective case series, we described six different NIDHRs of variable severity after intravitreal anti-VEGF. While three of our patients showed a relatively mild reaction with a maculopapular rash (Patient 2 and 5) or a pruritic erythematous swelling of both ankles (Patient 6), the three others of them had a more severe reaction ranging from palpable purpura (Patient 4) to inflammatory dermatosis either with localized pruriginous erythema (Patient 1) or generalized erythrodema (Patient 3). Cutaneous side effects of variable phenotypes and severity have already been described in the literature following intravitreal bevacizumab, ranibizumab and aflibercept injections. Some of them where consistent with the ones we saw, as maculopapular rashes. Other types of reactions, different from the ones we described, have also been reported, such as de novo cutaneous lupus erythematosus, acute generalized exanthematous pustulosis, head and trunk papulopustular eruption, facial skin redness and itchy diffuse rash [12–17].

Onset of symptoms in our case series ranged from day 2 to 5 (Patients 1, 2, 4 and 6) to one month following the anti-VEGF IVT (Patients 3 and 5), lasting between 5 and 28 days after their onset. These times of onset were consistent with what is described in such late reactions in the literature, usually days or weeks after first drug administration, including after intravitreal anti-VEGFs [12–17, 22].

The systemic side effects after intravitreal anti-VEGFs injection may be explained by the penetration of the drug to the systemic circulation at a low proportion. Serum concentrations after intravitreal injection of ranibizumab, bevacizumab, and aflibercept have been reported at low levels of 0.060 nM, 0.668 nM and 0.068 nM, respectively [23]. However, even low serum levels might be enough in some patients to provoke NIDHR.

Skin biopsy was performed for Patients 3, 4 and 6, which actually helped us on determining the type of reaction: Patient 3 had an eosinophilic spongiosis dermatitis with negative immunofluorescence, Patient 4 had a leucocytoclastic vasculitis with IgA deposits, and Patient 6 had a superficial dermatitis with dermal swelling and granular C3 deposits in the vessel’s walls on direct immunofluorescence. All of these results suit well a type III hypersensitivity reaction, secondary to the accumulation of immune complexes (ICs) (antigen–antibody complexes) in the tissue. Similar reactions have already been described in the literature following systemic mAb administration: leucocytoclastic vasculitis has been related to infliximab (anti-tumor necrosis factor (TNF) mAb) [24], erythroderma secondary to tocilizumab (humanized anti-human interleukin 6 receptor (IL-6R) antibody) [25] and maculopapular rash after anti-TNF mAb [19]. In particular, systemic use of anti-VEGFs in oncology has also been reported to induce cutaneous adverse events, including unspecified skin rash, exfoliative dermatitis and acute and severe acne [26–28].

Based on the history, the clinical description and on the available skin biopsies for our patients, the described eruptions were very probably type III hypersensitivity reactions. In general, NIDHRs secondary to biologic agents are mostly type III hypersensitivity reactions [21].

Although the precise mechanisms underlying type III hypersensitivity reactions to biologic agents are not fully understood, the suggested pathophysiological mechanism postulated so far includes the production of anti-drug antibodies (ADAs) [29]. Immune-mediated adverse effects attributed to ADAs require the formation of ICs involving the therapeutic molecule and ADA. These ICs tend to form deposits at anatomical sites with diffuse capillary network leading to inflammatory response, responsible for the type III hypersensitivity reaction.

ICs are heterogeneous and vary with ADA concentrations, which influences the intensity of adverse effects. In case of low ADA concentration, no adverse effect may occur. In contrast, in case of high ADA concentration, a significant amount of ICs may form and induce the typical adverse effects. Furthermore, these ICs may be formed with IgM ADAs (non-specific immunoglobulins with low affinity to the antigen) or IgG (specific immunoglobulins with high-affinity to the antigen). Therefore, these adverse reactions may also occur after the first exposure to the biologic agent, as illustrate in Patient 5. However, in most cases one or more previous exposures are reported [20].

ADA titers may vary from over time, and from one drug exposure to the other. This might explain the sudden but often late appearance of the immune reaction, and the possibility of well tolerated re-exposure to the same drug [29].

However, in general the clinician will seek for a different treatment if available, thus reducing the risk of recurrence. As bevacizumab, ranibizumab, aflibercept and brolucizumab share some parts in their structure [4, 5], cross-reactivity may occur despite the use of another intravitreal anti-VEGF [30]. Indeed, ADAs can be directed against the shared part of their molecular structure [29]. Therefore, the change in anti-VEGF may not protect against the recurrence of a type III hypersensitivity reaction, as illustrated with Patients 1 and 3, who experienced recurrence in cutaneous symptoms after switch to another anti-VEGF.

The diagnosis of NIDHRs is sometimes difficult to establish, as the clinical manifestation and the time of onset are highly variable. In case of NIDHR suspicion secondary to intravitreal anti-VEGFs, we recommend a meticulous history and physical examination by a specialist (dermatologist, allergist or immunologist). Skin biopsy is indicated, and may help to differentiate the type of reaction. Patients 3, 4 and 6 underwent skin biopsies, which actually helped to establish the diagnosis.

Skin tests with antibodies (such as done with ranibizumab and bevacizumab for Patient 1) are often negative and not very helpful. This may be related to the precipitation of ICs into skin microvessels [31]. As the predictive value of skin tests is low in case of suspected type III hypersensitivity reaction, we don’t recommend using them systematically in routine practice.

Management of NIDHRs consists of topical and systemic corticosteroids. Antihistaminics are also used to reduce prolonged or late phase reactions.

In addition, avoidance of the incriminated medication in case of severe drug-related hypersensitivity reaction must be the rule. However, in case of isolated and mild cutaneous symptoms, treatment continuation might be an option, if there is an absolute necessity to carry on therapy [30].

Weaknesses of this study are related to its retrospective character, with non-standardized clinical work-up. This has led to partially missing skin biopsies, a variable follow-up duration, and missing photos. In addition, the numbers are small due to the rare incidence of NIDHRs after intravitreal anti-VEGF (rarer than endophthalmitis). However, these reported 6 cases may help to increase awareness of the problem within the ophthalmic community. It would be useful to collect multicentric international information on this topic in order to better describe and understand the problem. Although the causal relationship may be difficult to establish in some cases, the slowly increasing number of reported cases confirms that NIDHRs may occur after intravitreal anti-VEGF treatment.

In conclusion, this retrospective case series supports the hypothesis that intravitreal anti-VEGF may lead to NIDHR, with variable range of severity and phenotypes. According to our experience, cross-reactivity between bevacizumab, ranibizumab and aflibercept exists, but is not the rule. Therefore, switch between anti-VEGFs, although indicated, may not protect completely against recurrence. So far, there is no corresponding information for brolucizumab. A multidisciplinary management with dermatologist, allergist or immunologist is required. In particular, we recommend proceeding to a skin biopsy for each suspected case. In case of isolated mild cutaneous symptoms, continuation of the therapy is possible if absolutely needed. In case of associated systemic features, an alternative management of the disorder must be considered.

## Data and materials availability

The files were reviewed for extracting the data.
